# Supplementary figures and images for: Honey bee viruses in Serbian colonies of different strength
Source: PeerJ. 2018 Nov 14;6:e5887. doi: 10.7717/peerj.5887 (PMC6240340; doi:10.7717/peerj.5887)

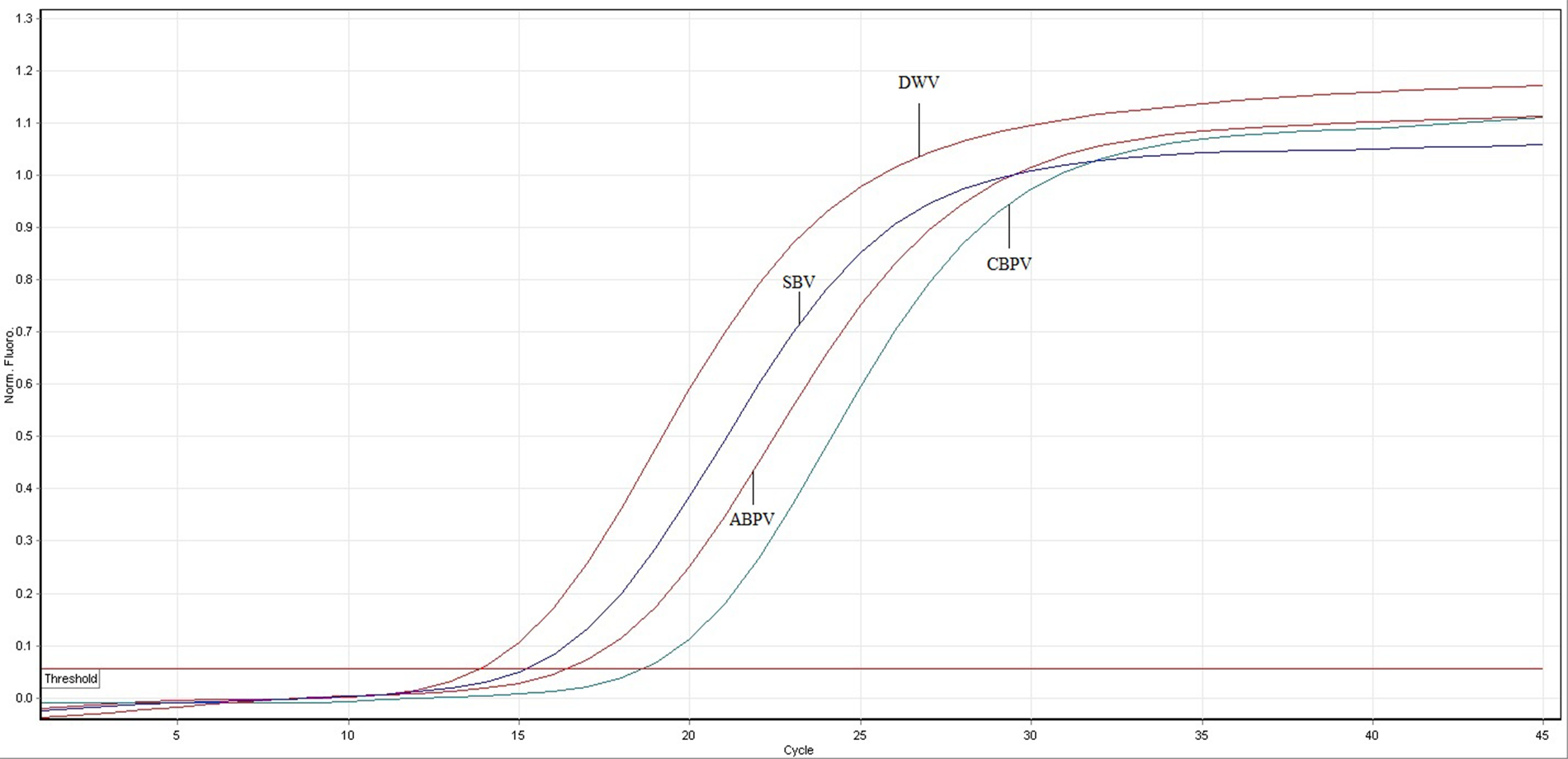

Supplement: Figure S1 [file peerj-06-5887-s002.png]

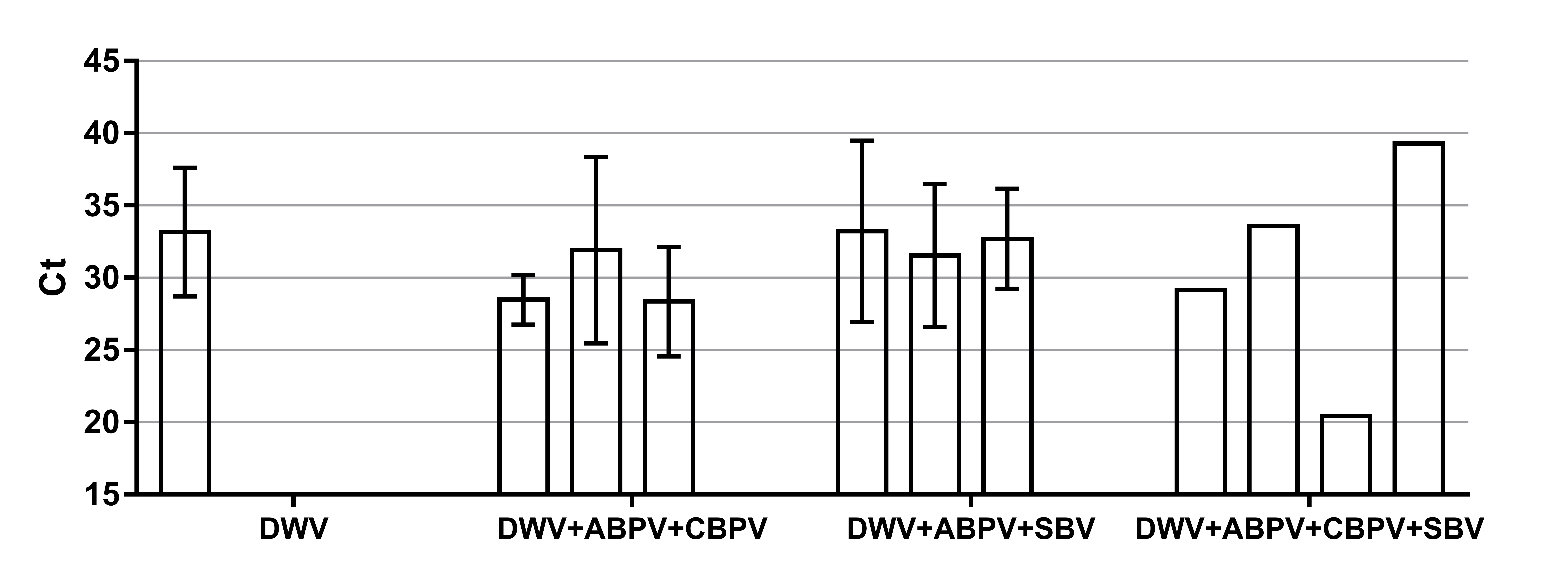

Supplement: Figure S2 [file peerj-06-5887-s003.png]
