# Supplementary material for: Honey bee viruses in Serbian colonies of different strength
Source: PeerJ. 2018 Nov 14;6:e5887. doi: 10.7717/peerj.5887 (PMC6240340; doi:10.7717/peerj.5887)
Supplement: Table S1 [file peerj-06-5887-s004.docx]

**Table S1.** Ct value ratios of viruses in double virus infections in colonies of different strength

| **Colony strength** | **Double virus infections** | | | | | |
| --- | --- | --- | --- | --- | --- | --- |
|  | **DWV/ABPV** | **DWV/CBPV** | **DWV/SBV** | **ABPV/CBPV** | **ABPV/SBV** | **CBPV/SBV** |
| **Strong** | 1.0809 | 1.0291 | 1.0489 | 0.9520 | 0.9703 | 1.0192 |
| **Medium** | 0.9908 | 1.0614 | 0.9657 | 1.0713 | 0.9746 | 0.9097 |
| **Weak** | 1.0637 | 1.1264 | 0.9337 | 1.0589 | 0.8778 | 0.8289 |
